# Iron Supplementation at the Crossroads of Nutrition and Gut Microbiota: The State of the Art

**DOI:** 10.3390/nu14091926

**Published:** 2022-05-04

**Authors:** Ana M. Puga, María de Lourdes Samaniego-Vaesken, Ana Montero-Bravo, Mar Ruperto, Teresa Partearroyo, Gregorio Varela-Moreiras

**Affiliations:** Grupo USP-CEU de Excelencia “Nutrición para la vida (Nutrition for Life)”, ref: E02/0720, Departamento de Ciencias Farmacéuticas y de la Salud, Facultad de Farmacia, Universidad San Pablo-CEU, CEU Universities, Urbanización Montepríncipe, 28660 Boadilla del Monte, Spain; anamaria.pugagimenezazca@ceu.es (A.M.P.); l.samaniego@ceu.es (M.d.L.S.-V.); amontero.fcex@ceu.es (A.M.-B.); mmar.rupertolopez@ceu.es (M.R.); t.partearroyo@ceu.es (T.P.)

**Keywords:** iron supplementation, iron fortification, gut microbiota, microbiome, iron deficiency, iron-deficiency anemia, micronutrient powders, iron status

## Abstract

Gut microbiota has received significant attention owing to its decisive role in human health and disease. Diet exerts a significant influence on the variety and number of bacteria residing in the intestinal epithelium. On the other hand, as iron is a key micronutrient for blood formation and oxygen supply, its deficiency is highly prevalent worldwide. In fact, it is the most common cause of anemia and thus, iron supplementation is widespread. However, there is concern due to some potential risks linked to iron supplementation. Therefore, we have reviewed the available evidence of the effects that iron supplementation exerts on the gut microbiota as well as its potential benefits and risks. The compiled information suggests that iron supplementation is potentially harmful for gut microbiota. Therefore, it should be performed with caution, and by principle, recommended only to individuals with proven iron deficiency or iron-deficiency anemia to avoid potential adverse effects. In any case, large and long-term population studies are urgently needed to confirm or refute these results, mainly focused on vulnerable populations.

## 1. Introduction

In the past decade, the gut microbiota has received significant research attention owing to its decisive role in human health and disease [1,2]. Previous studies have shown that diet exerts a significant influence on the variety and number of bacteria residing in the intestinal epithelium [3]. Gut microbiota is described as a complex, dense, and dynamic microbial population/ecosystem that inhabits the human gut and can markedly influence the health and disease status of their host [4]. When referring to the “microbiome” this term is sometimes used interchangeably with the former, but it encompasses the entire gene pool of gut bacteria, which numbers up to 150 times more than the genes in the human genome [5]. Characterization of this immensely diverse ecosystem is the first step in elucidating its role in health and disease conditions. The microbiota is mainly represented by anaerobic bacteria, but also includes archaea, yeasts, and other eukaryotes, the majority of them being commensal or mutualistic microorganisms. While more than 50 bacterial phyla have been described, the gut microbiota is dominated by *Firmicutes*, *Bacteroidetes*, *Actinobacteria*, and *Proteobacteria* [6]. Interestingly, it has also been regarded as an essential “organ” that provides nourishment, regulates epithelial development, and induces innate immunity, establishing a symbiotic relationship that results in a functional intestinal barrier and physiological homeostasis [7].

The prenatal gastrointestinal tract is sterile because of the protective immunological placental barrier and microbial colonialization develops progressively when environmental contact first occurs at birth. Consequently, the intestinal microbiota is derived at least in part from the mother during the birthing process and is modified subsequently by factors such as diet, antibiotic use, host genetics and other environmental factors [8]. During the first days of life, the main bacteria colonizing the infant gut are *Bifidobacterium* and *Clostridium* [9]. Feeding type is one of the main features determining early microbial colonization. In fact, there is widely available literature describing the differences in gut microbial composition between breastfed and formula-fed infants [10,11] and some authors affirm that there is no singular optimal gut microbiota composition since it is different for each individual [12].

The acquired knowledge and research underlying the characterization of these microorganisms and their crucial functions has greatly increased over the past two decades. In human gut microbiota, three basic bacterial enterotypes have been described: (1) genus *Prevotella*, which is considered mostly anti-inflammatory and thus protective, (2) genus *Bacteroides*, which is mainly pro-inflammatory and possibly associated with increased metabolic syndrome risk and other pathological conditions and, (3) genus *Ruminococcus*, with lower biological significance [13]. The gut microbiota may benefit the host through many physiological functions such as strengthening the integrity of the intestinal barrier, energy recovery and vitamin production, protecting against pathogenic agents, and regulating host immunity [4,14]. However, these mechanisms may be disrupted as a result of an altered microbial composition, known as dysbiosis, which in turn could be linked to the pathogenesis of various common metabolic disorders including obesity, type 2 diabetes, cardio-metabolic diseases, non-alcoholic liver disease, inflammatory bowel disease, undernutrition, and cancer [15].

As previously stated, diet plays a crucial role in the modulation of gut microbiota composition [16]. Studies have shown that microbiota from individuals living in the pre-industrial era was mainly dominated by *Prevotella*, whose presence is scarce in industrial and Western countries, where *Bacteroides* is the predominant genus [16,17,18]. Likewise, people following vegetarian and vegan diets show a greater presence of bacteria from the enterotype *Prevotella* [19]. Bolte et al. [3] showed that dietary patterns that included legumes, breads, fish, and nuts were associated with a lower abundance of clusters of opportunistic bacteria, being those a pathway for the synthesis of endotoxins and inflammatory markers in human stool. They also observed higher abundances of commensals such as *Roseburia*, *Faecalibacterium*, and *Eubacterium* spp. with the consumption of nuts, oily fish, fruits, vegetables, cereals, and red wine across all cohorts analyzed [3]. These bacteria are known for their anti-inflammatory effects in the intestine through fermentation of dietary fiber to short-chain fatty acids (SCFA) [8]. The Mediterranean diet is traditionally rich in these foods and has been associated, in retrospective studies, with a lower inflammatory bowel disease risk [20].

The latest evidence reviewed by Merra et al. [21] suggests that gut microbiota of subjects that follow a Mediterranean diet, rich in polyphenols, polyunsaturated fatty acids (PUFA), ω-3 and fiber is significantly different from subjects that follow a Western diet pattern. Fiber is an essential substrate for the production of SCFA through microbial fermentation, which plays a vital role in modulating the immune response and serves as a primary energy substrate for intestinal epithelial cells [22]. These findings are in line with those from human cohorts that consume adequate or large amounts of dietary fiber and present a lower incidence of inflammatory disease, including ulcerative colitis and colorectal cancer [23]. The Western diet pattern has been associated with an increased risk of ulcerative colitis and flare occurrence in inflammatory bowel disease. The typical Western diet is characterized by higher amounts of processed food, red meat, fat, sugar, emulsifiers, and additive exposure, as well as reduced amounts of fiber, fruit, and vegetables [24]. In inflammatory bowel disease, the homoeostasis between the gut microbiota and the intestinal immune response is lost [3]. Furthermore, the gut microbiota may also affect systemic immune mechanisms implicated in an increasing number of immune-mediated inflammatory diseases, including diabetes, arthritis, and systemic lupus erythematosus, presumably by altering intestinal barrier permeability, modifying self-antigen integrity, mimicking epitopes, and modulating cell apoptosis mechanisms [25,26]. Gut dysbiosis and the associated inflammation have also been related to certain types of cancer (i.e., colorectal) [27] as well as several cardiometabolic disorders [28], asthma [29], or type 1 diabetes mellitus [30].

On the other hand, iron is a key micronutrient for blood formation and oxygen supply, being essential for the synthesis of hemoglobin. In addition, it enables numerous metabolic reactions as an enzymatic cofactor, participating in DNA synthesis and methylation and oxygen transport [31]. Its deficiency is highly prevalent worldwide and it is the most common cause of anemia, a condition characterized mainly by low blood hemoglobin concentration, which decreases the capacity of the blood to carry oxygen to tissues [32]. The prevalence of anemia in women of reproductive age (15–49 years) has been estimated to be 33% of the world’s population [33], namely in regions such as Africa, South-East Asia, and the Eastern Mediterranean, which are reported to have the highest prevalence (35%) [33]. Moreover, currently, it has been estimated that the prevalence of iron deficiency (ID) amongst European women is about 10–32% and iron-deficiency anemia (IDA) 2–5% [34], representing one of the main nutritional deficiency disorders and affecting both industrialized nations as well as developing countries [35]. The recommended iron intakes for women of fertile age ranges from 14.8 to 20 mg/d depending on the country [36]. In Spain, Daily Recommended Intakes (DRI) of iron for women of childbearing age is 18 mg/day and 10 mg/day for men and postmenopausal women [37]. Dietary surveys conducted in European countries described that large segment of children and women have dietary iron intakes below these recommendations. Results from representative dietary surveys in Spain, such as the ANIBES study, found that a high proportion of the population did not meet the iron DRI [38]. The total median dietary iron intake was 9.8 mg/d for women and 11.3 mg/d for men (18–64 years in both cases). The highest intakes were observed among children (12.2 mg/day; 9–12 years), adolescents (13.3 mg/day; 13–17 years), followed by adults (13.0 mg/day; 18–64 years) and the elderly (12.7 mg/day; 65–75 years) populations. Prevalence of adequacy for iron intakes as assessed by European Food Safety Authority (EFSA), whose criteria were higher than for the Spanish recommended iron intake values. In all age groups, females showed lower adequacy than males for both criteria, 27.3% and 17.0% vs. 77.2% and 57.0%, respectively.

Iron requirements increase during pregnancy and dietary sources cannot always prevent its deficit; additionally, maternal anemia, especially in the second trimester, has been associated with negative outcomes, including maternal mortality, preeclampsia, low birth weight and premature birth [39]. Therefore, during pregnancy, supplementation recommendations in Europe vary from 27 mg/day [40] to 40 mg/day [41], whereas the EFSA recommends not to increase iron intake based on the assumption of the increase in iron absorption over this period [36]. On the other hand, the World Health Organization (WHO) recommended the universal iron supplementation of pregnant women [42]. It is also acknowledged that the widespread use of iron supplements during pregnancy in some European countries has lowered the prevalence of ID in pregnant women [35].

However, there is concern related to some of the potential risks linked to iron supplementation such as the generation of reactive oxygen species that can damage the cell’s organic components. In addition, in the recent years, there has been a growing worry about non-transferrin-bound iron (NTBI), which is an important biomarker related to the iron loading status of patients with certain diseases. NTBI is a highly toxic form of iron that is capable of generating free radicals and can lead to oxidative damage of various tissues. Therefore, it is critical to quantify NTBI in blood to prevent high-iron concentrations that can lead to toxicity and organ failure [43]. Moreover, it is important to consider that a large portion of enteral nonheme iron is unabsorbed and is potentially available as an essential nutrient for bacteria inhabiting in the colon. In fact, it is known that potential pathogens like *Escherichia coli* (*E. coli*) and *Salmonella* or *Shigella* are some of the most siderophilic species [44,45], whereas on the other hand, beneficial bacteria such as *Bifidobacteria* or *Lactobacillus* have low iron requirements [46] and provide an important “intestinal barrier effect” against pathogen colonization [47]. Moreover, iron-rich environments can enhance the proliferation of other pathogenic microorganisms like the malaria protozoa, *Plasmodium falciparum* [48].

Malnutrition is a global health epidemic, with 240 million children worldwide wasted and stunted, including 45 million under the age of five [49]. Undernourished children present an increased vulnerability to infections and thus a higher death risk, even from diarrheal complications [50,51]. Gut microbiota plays an important role in undernutrition due to dysbiosis and intestinal immaturity (i.e., developmental immaturity of bacterial communities relative to age) [52,53]. In fact, in infants, the amount of iron available in the gut influences the microbiome (highly mutable and whose establishment takes place in the first months after birth). A low intraluminal iron concentration favors the growth of the preferred *Lactobacillus* organisms, while high iron fosters a shift in the microbiota toward potentially pathogenic *Bacteroides* and *E. coli* as early as 1 week of postnatal age [44,54]. Health programs focused on vulnerable children populations involve the use of micronutrient supplements, i.e., vitamins and minerals, which have been demonstrated to improve growth and reduce mortality [55,56]. Specifically, up to a few years ago, the WHO advised the provision of an oral supplementation with ferrous sulphate for children living in areas with high ID prevalence [57,58]. However, this recommendation has been changed owing to the potential risk of increased hospitalizations and mortality from infections due to iron supplementation [58,59].

Iron supplementation and fortification programs are widely used strategies for the prevention and control of ID and IDA [60]. In addition, malaria is an important cause of ID in African children [61]. Therefore, in endemic areas (mainly, in African countries), the use of iron supplementation (by means of tablets, syrups or micronutrient powders (MNPs)) is the primary intervention to cope with ID and IDA. However, there are important concerns regarding their safety and efficacy in these countries since they may predispose individuals to malaria and other infections; in addition, these supplements could be poorly absorbed [62,63], thus leading to alterations in gut microbiota. 

Furthermore, in rural African populations with high levels of inflammation and infection, iron absorption in the gut is likely to be even lower since inflammation increases circulating hepcidin (the main iron regulator, which reduces dietary iron absorption through the binding and degradation of ferroportin, an iron efflux protein at the basolateral membrane of the enterocytes) [64]. However, ID may have potential benefits and for instance, ID and IDA have been proposed to play a protective role against malaria infections [65]. Therefore, nutritional supplementation in endemic areas is controversial since the provision of iron may increase malaria morbidity and mortality [62,66].

Thus, it has been hypothesized that despite iron supplementation is necessary in many cases for the prevention or treatment of ID and IDA, high levelof iron in the gut may have unfavorable effects on the individual’s gut microbiota. Therefore, in the present review, we aim to shed light on the latest available evidence of the effects that iron supplementation exerts on gut microbiota, and the potential benefits and risks of its supplementation with a special focus on vulnerable population groups. For a better understanding and follow-up, we have structured this review based on the population group included in each intervention, highlighting the different iron formulations employed and their effects on bacterial microbiota as well as other biochemical parameters evaluated.

## 2. Materials and Methods

### 2.1. Search Methods

Intervention studies from database initiation up to and including 15 February 2021 were searched using the following bibliographic online databases: MEDLINE, PubMed, and Scopus, using the subsequent search terms: “iron”; “supplementation”; “fortification”; “microbiota”; “microbiome” AND “intervention”. Articles were restricted to those conducted in humans and published in English or Spanish.

### 2.2. Selection Criteria and Eligibility

Eligible populations included both men and women of all age groups and were classified as: (i) infants and toddlers from 0 to 36 months of age, (ii) children and adolescents from 3 to 18 years; and (iii) adults older than 18 years. Articles were eligible if they included iron supplementation either alone or in combination with other vitamins or minerals, disregarding if the participants suffered from ID/IDA or not. For the evaluation of the alterations in gut microbiota profiles, bacteria found in fecal samples, stool, and blood biomarkers were selected as the main outcomes.

## 3. Intervention Studies Performed in Infants and Toddlers

The first studies that determined the impact of iron on the microbiota date back to the 1980s and were conducted in infants (Table 1). These studies aimed to analyze the effect of iron on neonatal gut microbiota over the first week [67] or the first three months of life [68]. Thus, the first study compared the microbiota of stool samples of infants (*n* = 23) from Utrecht (The Netherlands) who received a cow’s milk preparation supplemented with iron (5 mg/L), an unfortified cow’s milk preparation or breast milk in the prior 7 days of life. Results from this study revealed that infants fed with breast milk showed high level of *Bifidobacteria* and low levels of *Enterobacteriaceae*, *Bacteroides*, and *Clostridia*. Regarding the infants fed with cow’s milk formulations, those who received iron-fortified milk showed high levels of *Enterobacteriaceae*, *Bifidobacteria* and putrefactive bacteria such as *Bacteroides* and *Clostridia*, whereas those who received unfortified milk presented a slow increase in both *Enterobacteriaceae* and *Bifidobacteria* counts. These results seemed to indicate that iron may favor gut colonization and low iron content enhances the resistance of the neonatal gut to colonization [67]. Later on, the same authors extended the study up to the first 12 weeks of life of the initial cohort of infants (*n* = 23) with fecal samples collected weekly. This study confirmed that *Bifidobacteria* was predominant in infants fed with breast milk, as observed in the previous study, and that other bacteria were rarely present. Moreover, infants who received iron-fortified cow’s milk formulations developed a complex aerobic and anaerobic microbiota with low presence of *Bifidobacteria* and high counts of *E. coli* and *Clostridia*. Finally, the administration of unfortified cow’s milk was associated with a bacterial gut flora that resembles the flora observed in children fed with breast milk. Therefore, this may be associated with a protective effect of using this milk against gut colonization by pathogenic microorganisms [68].

Owolabi et al. [69] conducted an open-label randomized study to analyze the effect of a multi-nutrient-fortified dairy based drink on gut bacteria. This study was carried out in malnourished Nigerian toddlers (*n* = 184, aged 12 to 36 months) with mild–moderate anemia. Participants were randomly assigned to receive 200, 400 or 600 mL of a multi-nutrient-fortified dairy-based drink (containing 2.24, 4.48 and 6.72 mg of elemental iron, respectively) per day for six months. Blood samples were collected at baseline and endline to determine hemoglobin, ferritin, and C-reactive protein (CRP). Likewise, gut microbiota composition was analyzed from fecal samples at the same time points. Supplementation significantly reduced ID and IDA prevalence, even with the formulation with lower iron concentration. Regarding gut microbiota, *Enterobacteriaceae* relative abundance decreased over time, with the highest relative abundance in infants receiving 400 mL and the lowest in infants receiving 600 mL. However, a relative abundance of *Bifidobacteriacea* did not differ between dose groups and slightly decreased over time. Finally, pathogenic *E. coli* also decreased at the end of the intervention and no differences were detected between groups. Therefore, the authors concluded that multi-nutrient-fortified dairy-based drinks reduced anemia in a dose-dependent manner, without promoting the proliferation of potentially pathogenic microorganisms in the gut [69]. The lack of adverse effects on the gut microbiota was attributed to the low levels of relatively high bioavailable iron per serving (iron sulphate, vitamin C, and no phytic acid). In any case, it is important to consider that the highest intake volumes (600 mL) could be rather difficult to drink for small children and this may be an explanation for the lack of differences observed between groups.

Complementary feeding between 6 and 24 months of age is of key importance for nutritional and developmental reasons due to the shift from exclusive milk-feeding to family meals. In addition, complementary feeding is the critical period when ID and IDA are more prevalent. These problems are often addressed through nutritional intervention programs [70]. Thus, Krebs et al. [71] compared iron status in exclusively breastfed infants from Minneapolis (*n* = 45, 5 months aged) who randomly received pureed meats, iron and zinc-fortified cereals or iron-only-fortified cereals (containing 1.0, 7.8 or 6.2 mg of iron, respectively), from 6 to 9 months of life. Fruits, vegetables, teething biscuits, and unfortified cereals and other finger foods were permitted ad libitum. Mothers received guidelines on the amounts of complementary foods suitable for the infants and recommendations for gradually increasing servings (from one serving per day by 7 months (i.e., 15 g of dry cereal or 71 g jar of meat) to two servings per day by 9 months). Biochemical blood parameters were analyzed (serum ferritin, CRP, and serum transferrin receptor concentrations as well as hematological indexes). Furthermore, in a subsample of infants, monthly stool samples were collected starting at 5 months of age as baseline for microbiome analysis by means of PCR. Approximately one quarter of the studied participants showed low serum ferritin levels and 36% were mildly anemic without significant differences among groups. Both the feeding group and the amount of iron intake (greater in those volunteers who received cereals) were significantly associated with longitudinal changes in the enteric microbiome. Bacteria from phylum *Firmicutes* significantly increased in volunteers who received meat vs. volunteers fed with cereals. Moreover, *Enterobacteriaceae* decreased in all groups (especially in meat volunteers) although no significant differences were found. Likewise, a significant decrease was observed in *Lactobacillales* in participants receiving only iron-fortified cereals with no changes over time in the other groups. No evidence was found relating iron fortification to a greater abundance or diversity of pathogenic microorganisms. In any case, these results must be considered with caution due to the limited sample size [71].

The use of MNPs is a common strategy to prevent ID, IDA, or other micronutrient-related deficiencies, mainly at home in low-resource settings. In this sense, several studies evaluated the impact of these formulations on gut microbiota. The majority of the available studies have been carried out in low-income countries, especially in malaria-endemic areas, mainly in Kenya [72,73,74,75]. In this sense, Tang et al. [72] performed a double-blind randomized controlled trial with the aim of evaluating the effects of iron on gut microbiota and inflammatory status in non- or mildly anemic Kenyan infants. Volunteers were randomized into three groups (*n* = 15 each): MNPs+Fe (containing 12.5 mg iron/day and 5 mg zinc/day), MNPs-Fe (the same MNPs without iron) and control group (MNPs without micronutrients). Supplementation lasted for 3 months (from 6 months up to 9 months of age) and mothers were asked to add the entire content of the sachet to one meal. Stool samples were collected at the baseline and at the end of the intervention and the microbiome was profiled. Fecal calprotectin, a fecal biomarker of gut inflammation, and other systemic blood markers of inflammation (α 1-acid glycoprotein (AGP), CRP, tumor necrosis factor-α (TNF-α), interleukin (IL)-6 and IL-8) were also determined as well as iron status. At the end of the intervention, a decrease in the relative abundance of *Bifidobacterium* was observed in MNPs+Fe group and in controls but not in MNPs-Fe group, whereas the relative abundance of *Escherichia* significantly decreased in MNPs-Fe and controls, but not in the MNPs+Fe group. The obtained results were consistent with previous studies [67,68], confirming that iron supplementation negatively affect gut colonization by beneficial microorganisms and attenuate the decrease in pathogenic strains. Despite these findings, no differences among groups were observed in the analyzed inflammatory biomarkers. According to the authors’ opinion, this lack of significance in inflammatory biomarkers could be affected once again by the relatively small sample size, at least partially [72].

The combination of iron with other micronutrients (vitamins and minerals) may lead to differences in the impact of supplementation on gut microbiota. A study conducted in Pakistan analyzed the effect of MNPs containing vitamins, iron, and zinc in bacterial diversity and microbiota interactions. The study included 80 children, which provided 160 paired stool samples at 12 and 24 months of age (*n* = 24 children of the control group, *n* = 29 children receiving MNPs, and *n* = 27 children receiving MNPs with zinc). Volunteers were part of a larger cluster randomized controlled trial [76] and supplementation lasted from 6 to 18 months of age. The caregivers were instructed to mix one sachet of MNPs per day with the child´s regular food. All participants, including those of the control group, received health promotion messages and recommendations for feeding practices. MNPs contained vitamins A as retinol (300 μg), C (50 mg), cholecalciferol (vitamin D_3_) (5 μg), folic acid (150 μg), and microencapsulated iron (12.5 mg), with or without zinc (10 mg) [76]. Microbiota diversity was analyzed in terms of richness (number of operational taxonomic units), evenness, as well as differences in bacterial composition and microbial interactions. Results showed that supplementation without zinc was related to different eukaryotic communities, with an increased prevalence of protozoa and fungi, including species with known potential to cause symptomatic infections and disrupt gut microbiome. In contrast, zinc supplementation ameliorated these increases and decreased the prevalence of *Toxoplasma* and the overall richness of protozoa. These results confirmed the impact of MNPs in infant microbiota, suggesting the possibility of the existence of disruptive effects driven through the promotion of specific microorganisms during the early steps of microbiome development [77]. In this regard, combination of different minerals (for example, iron and zinc) could be of interest, although larger studies are needed to confirm the clinical significance of these findings.

Another study conducted in Kenyan infants compared the effect of two different iron formulations (sodium iron ethylenediaminetetra-acetate, NaFeEDTA vs. ferrous fumarate) on gut microbiota and intestinal inflammation in 115 infants aged 6 months. The study was carried out in two stages. During the first period, infants randomly received MNPs containing or not containing 2.5 mg iron/day as NaFeEDTA (that is, ferric sulphate bound to a chelator) for 4 months. Then, in the second period, different infants received different MNPs containing or not containing 12.5 mg iron/day as ferrous fumarate. Biochemical parameters were determined at the baseline and at the end of the intervention and included hemoglobin, ferritin, CRP, and some cytokines (several IL and TNF-α). Stool samples were collected at the baseline and after 3 weeks and 4 months of supplementation. Then, gut microbiome profile (by means of 16S rRNA pyrosequencing and targeted qPCR) and fecal calprotectin were analyzed. At the end of the intervention, significant differences were found in the gut microbiome of infants who received MNPs with NaFeEDTA vs. those who received MNPs with ferrous fumarate. In fact, ferrous fumarate caused greater modifications in gut microbiome as well as more intestinal inflammation, probably due to the fact that iron dose was fivefold that of NaFeEDTA (12.5 mg vs. 2.5 mg), although the relative concentration of ferrous or ferric iron in the gut after ingestion was unknown. Hence, ferrous fumarate MNPs resulted in a lower presence of *Bifidobacterium*, higher levels of fecal calprotectin and higher rates of treated diarrhea, NaFeEDTA MNPs showed greater effect on *Escherichia*/*Shigella*, the ratio *Enterobacteria* to *Bifidobacteria* and pathogenic *E. coli*. Regarding infants who received MNPs without Fe, the gut microbiome analysis revealed an increase in *Faecalibacterium* and *Prevotella* and a decrease in *Enterobacteriaceae*, compared to baseline [73]. According to the authors´ opinion, Fe supplementation should not be used indiscriminately in infants, but should be targeted only to infants with ID, coupled with proper diarrhea protection.

Another researched strategy to increase MNPs formulations safety is its combination with galacto-oligosaccharides (GOS). These prebiotics enhance the growth of *Bifidobacteriaceae* and *Lactobacillaceae* and increase colonic synthesis of SCFAs that decrease intestinal lumen pH, thus leading to a reduction in enteropathogens growth. Owing to their safety, GOS are often added to commercial infant formula [78]. Paganini et al. [75] conducted a study on155 Kenyan infants aged 6.5–9.5 months who were randomly divided into three groups to receive daily: (i) MNPs without iron, (ii) MNPs with 5 mg of iron (2.5 mg as NaFeEDTA and 2.5 mg as ferrous fumarate) and (iii) the same MNPs than group ii with 7.5 g of GOS. Supplementation lasted 4 months. Serum ferritin, soluble transferrin receptor, CRP and AGP were determined in blood samples at the baseline and at the end of the study. Likewise, gut microbiota was characterized from fecal samples collected at the baseline and after 3 weeks and 4 months of intervention. No differences were observed neither at baseline nor at the end of the intervention in the analyzed inflammatory markers. Concerning microbiota analysis, iron alone had a greater impact than iron with GOS or compared to controls. At the end of the intervention, iron alone compared to controls showed lower concentrations of genus *Lactobacillus* and *Bifidobacterium* and greater abundances of the order *Clostridiales* and family *Enterobacteriaceae*, but no differences in phylum *Bacteroidetes*. When comparing iron alone vs. iron with GOS, lower abundances of genera *Lactobacillus* and *Bifidobacterium* were determined and higher concentrations of order *Clostridiales*, but no differences in *Enterobacteriaceae* or *Bacteroidetes* [75]. Therefore, the obtained results confirm the potential beneficial effects of prebiotics in reducing the adverse effects of iron in infants gut microbiota.

Antibiotics are one of the most commonly prescribed drugs to infants, especially in low-resource settings. Due to the strong modifications, they cause in gut microbiota, in a subgroup of the previous study, the effect of antibiotics in gut microbiota was evaluated when given MNPs containing or not containing iron [74]. This way, the study included 28 Kenyan infants between 8 and 10 months of age who received: antibiotic and MNPs with 2.5 mg of iron (Ab+Fe+), antibiotics and MNPs without iron (Ab+Fe−), no antibiotics and MNPs with 2.5 mg of iron (Ab−Fe+) or no antibiotics and no iron MNPs (Ab−Fe−). Antibiotic treatment lasted 5 days, whereas supplementation with MNPs, 40 days. Fecal samples were collected at the baseline and after 5 days of antibiotic treatment and after 10, 20 and 40 days of supplementation. The main outcomes of this study were fecal calprotectin and microbiome characterization by means of qPCR. Results revealed large differences in gut microbiota composition amongst infants receiving antibiotics with iron vs. those who did not. Thus, infants from the Ab+Fe+ subgroup showed a decrease in abundances in *Bifidobacterium*, whereas an increase was shown in those from the Ab+Fe− subgroup. Moreover, in the latter subgroup, there was a decrease in pathogenic *E. coli* while in the Ab+Fe+ there was an increase in *Clostridium difficile*. Finally, diarrhea prevalence was higher in the Ab+Fe+ subgroup than in the Ab+Fe− or in the Ab−Fe+ [74]. Therefore, iron may reduce antibiotic efficacy against enteropathogens, and this combination may also have detrimental effects related to beneficial bacteria such as *Bifidobacterium*. Consequently, supplementation with MNPs with iron should be carried out with caution or even temporary discontinuation in the case of infants that require antibiotic therapy. In any case, large intervention trials are needed to confirm these findings.

Lipid-based oral nutrient supplements (LONS) are commonly used for the management of acute severe undernutrition, mainly in emergency settings.LONS. LONS are products where lipids provide the majority of energy but also include proteins, carbohydrates and micronutrients embedded in edible fats. The available evidence to date suggests that LONS are effective in aiding in the recovery from moderate acute undernutrition, lowering the risk of deterioration into severe undernutrition, and improving weight gained [79]. Nevertheless, the effect of LONS on gut microbiota is not well-understood. In this regard, an intervention study was performed in healthy Malawian infants (*n* = 160) aged 6 months. The participants were randomly assigned into four different intervention schemes. Infants in the control group did not receive any supplementary food during the primary follow-up period and were supplemented with 71 g/day of a micronutrient-fortified corn-soy blend (5.46 mg/day of iron) afterwards for 6 months. The infants in the remaining groups received 71 g/day of micronutrient-fortified corn-soy blend (5.46 mg/day of iron), 54 g/day of micronutrient-fortified LONS with milk protein base (6 mg/day of iron), or 54 g/day of micronutrient-fortified LONS with soy protein base (6 mg/day of iron). Stool samples were collected at baseline and after 6 months of intervention and qPCR was used to characterize gut microbiome. Dietary supplements did not lead to changes in bacterial diversity or colony counts between intervention groups. However, modifications in microbiota composition in the total study population were detected with changes that seemed to shift towards an adult-type profile. Specifically, lower counts of *Bifidobacterium infantis*, *Bifidobacterium lactis* and *Bibidobacterium longum* were found, coupled with higher counts of total bacterial, *Bifidobacterium genus* and *Bifidobacterium catenulatum*. Moreover, *Staphylococcus aureus* decreased over time in the studied infants [37]. Subsequently, this intervention was extended up to 12 months. Infants from the control group, after the primary follow-up period, received supplementation with 71 g/day of a micronutrient-fortified corn-soy blend (5.46 mg/day of iron). Infants from the other groups maintained their supplementation rationale. The microbiota of paired stool samples (*n* = 213) was analyzed at 6 months of age (baseline) and at 18 months of age (i.e., 12 months of supplementation). No significant differences were observed among intervention groups, although participants supplemented with soy LONS seemed to have higher counts of some *Lactobacillus* strains. *Bifidobacterium longus* was the most abundant species at both ages, but mainly at baseline. *Bifidobacteriaceae* and *Enterobacteriaceae* significantly decreased with age, whereas *Prevotella* and *Faecalibacterium* significantly increased. *Salmonella*, *Shigella* and *Escherichia* were found but no differences were stated between groups or time points [80]. These results differ from those obtained in a similar study [73]. These differences could be attributed to disparities in the environment and the characteristics of the study´s cohorts. In any case, in the latter study, differences in iron dose among groups were very small, which could justify the lack of alterations in gut microbiota after supplementation [80].

Iron supplementation is widely used in women of reproductive age. Likewise, periconceptional and pregnant women are often supplemented owing to the high prevalence of ID and IDA due to the increased need of this mineral during pregnancy. In this regard, a study evaluated the impact of prenatal and postnatal nutritional interventions on infant gut microbiota. Specifically, the study was conducted in Malawi involving pregnant women (*n* = 869) who received daily supplementation during pregnancy and until 6 months postpartum. Women were randomly stratified into three intervention groups: (a) iron and folic acid (60 mg and 400 μg daily, respectively); (b) 20 mg/d of iron, 400 μg daily of folic acid and 16 additional micronutrients or (c) the same micronutrients than (b) but as LONS with four additional minerals, proteins, and fat. Infants from the latter group received the same supplements as mothers from 6 to 18 months, whereas those groups (a) and (b) did not receive any supplements. The infant’s microbiome was characterized by means of PCR from fecal samples collected by mothers at 1-, 6-, 12-, 18- and 30-months age and microbiota diversity and maturation were selected as primary outcomes. No differences were found in primary outcomes between the intervention groups a and b; therefore, these groups were combined. Microbiota diversity was higher in the LONS group (intervention group c) at 18 months (12 months of infant supplementation) but not in the remaining time points. However, microbiota maturation was not affected by supplementation. Regarding the taxonomic profile, the most abundant genus after 1 month was *Bifidobacterium* (mainly *Bifidobacterium longum*) which gradually decreased until 30 months. On the other hand, *Prevotella* abundance (predominantly *Prevotella copri*) increased with age, becoming one of the most abundant species at the end of the intervention. Moreover, the *Streptococcus* abundance remained stable over time, whereas *Faecalibacterium* increased from 6 to 30 months. Interestingly, household wealth appeared to modify microbial diversity at 30 months. Thus, among children in households with greater assets, higher diversity in the LONS group (intervention group c) compared to the iron, folic acid additional micronutrients group (intervention group b) was found, whereas no differences were observed among children in poorer households. This could be attributed to the fact that, children with higher asset scores have better quality diets (in terms of dietary diversity) and are less likely to remain breastfed. The obtained results seem to suggest that infant’s socioeconomic status may play a role in the maintenance of the microbiota diversity 12 months post-supplementation [81].

Due to iron availability problems, as well as the associated adverse effects of iron supplements, several researchers are evaluating new drug delivery systems for oral iron administration. In this regard, iron hydroxide adipate tartrate (IHAT) seems to be a promising alternative. IHAT is different from conventionally used iron compounds since it does not require solubilization in the stomach prior to its uptake by the enterocytes as it is absorbed as whole nanoparticles by endocytosis [82]. This means that the unabsorbed portion of the compound that transits the gut, about 70% of all orally ingested iron, will remain nanoparticulated and therefore not soluble, and as such will lead to fewer gastrointestinal adverse effects (including pathogens growth or tissue inflammation) [83]. IHAT-GUT study was a double-blind, randomized, placebo-controlled clinical trial conducted in Gambian children aged 6–35 months [84]. It included 705 children with IDA who were supplemented for 12 weeks with: (1) ferrous sulphate (12.5 mg of elemental iron equivalent daily), (2) IHAT (20 mg elemental iron equivalent daily, the bioequivalent dose considering the bioavailability of IHAT relative to ferrous sulphate) or (3) placebo (around 30 mg of pharmaceutical-grade sucrose daily). The primary outcome was to analyze differences among subgroups in terms of diarrhea incidence and prevalence. Secondary outcomes were fecal microbiota diversity and profile, abundance of enteric pathogens, and fecal calprotectin, among others [84]. Results of IHAT-GUT clinical trial revealed that iron supplementation did not significantly affect gut microbiota. In fact, volunteers’ age was the main factor that determined the overall bacterial composition of fecal samples. Furthermore, this study showed the development of a *Prevotella*-rich gut microbiota. This genus, as previously stated, is characteristic of non-industrial countries, whose diet is rich in fiber. It is important to highlight that this study provided a detailed analysis of microbiota of children during the first 40 months of life, a critical timeframe in human´s gut microbiota development [85]. According to the authors, further studies are needed to deepen into the characteristics of *Prevotella*-rich gut microbiotas, which are often scarcely studied and poorly represented in Western populations.

To date, most studies that focused on analyzing the impact of iron supplementation on gut microbiota in infants and toddlers have been performed in low-resource settings. In fact, evidence in developed countries is scarce. In this context, a study conducted in Swedish infants aged 6 months evaluated the effect of iron supplementation on gut microbiota. Thus, iron-sufficient infants were randomly assigned to receive low-iron-fortified formula (1.2 mg iron/day, *n* = 24), high iron-fortified formula (6.6 mg iron/day; *n* = 24) or no added iron formula with liquid ferrous sulfate supplementation (iron drops; 6.6 mg iron/day; *n* = 24). Total iron intake was 1.2, 6.4 and 5.7 mg/day in the low-iron, high-iron, and iron-drops group, respectively. Intervention lasted 45 days and stool samples were collected at baseline and at the end of the supplementation period. This research found that consumption of high-iron formula was significantly associated with a decrease in the relative abundance of *Bifidobacterium* compared with low-iron formula (60% vs. 78%) after only 45 days of study. However, no differences were found regarding pathogenic bacteria growth. Concerning infants receiving iron drops vs. high-iron formula group, lower relative abundance of *Lactobacillus* spp. was significantly found in the former one (8% vs. 42%). Moreover, unexpectedly, a higher relative abundance of *Lactobacillus* spp. (42% vs. 32%) in the high-iron group compared with low-iron formula group was also significantly found, suggesting that the mode of iron administration has a direct impact on *Lactobacilli* colonization in the gut. Additionally, the relative abundance of *Streptococcus* was lower in infants receiving iron drops, whereas *Clostridium* and *Bacteroides* was greater in infants receiving the high-iron formula [41].

**Table 1:** Summary of the effects of the intervention studies performed in infants and toddlers on gut microbiota.

Study Subjects	Intervention	Effects on Gut Microbiota	Author and Year
Newborn babies (*n* = 23) from The Netherlands.	(1) Cow-milk preparation supplemented with iron (5 mg/L), (2) unfortified cow-milk preparation or (3) Breast milk in the prior 7 days of life.	-High levels of *Bifidobacteria* and low of *Enterobacteriaceae*, *Bacteroides* and *Clostridia* in infants fed with breast milk. -High levels of *Enterobacteriaceae*, *Bifidobacteria* and putrefactive bacteria such as *Bacteroides* and *Clostridia* in infants receiving iron-fortified milk. -Slow increase in both *Enterobacteriaceae* and *Bifidobacteria* counts in infants receiving unfortified milk.	Mevissen-Verhage et al., 1985 [67]
Newborn babies (*n* = 23) from The Netherlands.	(1) Cow-milk preparation supplemented with iron (5 mg/L), (2) unfortified cow-milk preparation or (3) Breast milk for 3 months.	-Predominant presence of *Bifidobacteria* in infants fed with breast milk with rare detection of other bacteria. -Low presence of *Bifidobacteria* and high counts of *E. coli* and *Clostridia* in infants fed with iron-fortified cow milk formulations -Bacterial gut flora similar to that of infants fed with breast milk in infants who received unfortified cow milk.	Mevissen-Verhage et al., 1985 [68]
Malnourished Nigerian toddlers (*n* = 184) aged 12 to 36 months) with mild–moderate anemia.	(1) 200, (2) 400 or (3) 600 mL (containing 2.24, 4.48 and 6.72 mg of elemental iron, respectively) of a multi-nutrient-fortified dairy based drink per day for six months.	-Decrease in *Enterobacteriaceae* relative abundance over time (highest relative abundance in infants receiving 400 mL and lowest in infants receiving 600 mL). -No differences in the relative abundance of *Bifidobacteriacea* between dose groups, with a slight decrease over time. -Decrease in pathogenic *E. coli* at the end of the intervention, without differences among groups.	Owolabi et al., 2021 [69]
Exclusively breastfed infants 5 months aged, from Minneapolis (*n* = 45).	(1) Pureed meats, (2) iron- and zinc-fortified cereals or (3) iron-only-fortified cereals (containing 1.0, 7.8 or 6.2 mg of iron, respectively) from 3 months.	-Significant increase of bacteria from phylum *Firmicutes* in volunteers who received meat vs. volunteers fed with cereals. -Decrease in *Enterobacteriaceae* (especially in meat volunteers), without significant differences among groups. -Significant decrease in *Lactobacillales* in participants receiving only iron-fortified cereals (no changes over time in the other groups).	Krebs et al., 2013 [71]
Non- or mildly anemic Kenyan infants of 6 months age (*n* = 45).	(1) MNPs+Fe (containing 12.5 mg iron/day and 5 mg zinc/day), (2) MNPs-Fe (the same MNPs without iron) and (3) control group (MNPs without micronutrients) for 3 months.	-Decrease in the relative abundance of *Bifidobacterium* in MNPs+Fe group and in controls but not in MNPs-Fe group. -Significant decrease in the relative abundance of *Escherichia* in MNPs-Fe and controls but not in the MNPs+Fe group	Tang et al., 2017 [72]
Infants aged 6 months from Pakistan (*n* = 80).	(1) MNPs with microencapsulated iron (12.5 mg) with zinc (10 mg) or (2) MNPs with microencapsulated iron (12.5 mg) without zinc or (3) control for 12 months.	-Increase of protozoa and fungi prevalence (including species with known potential to cause symptomatic infections and disrupt gut microbiome) in infants supplemented without zinc. -Zinc supplementation ameliorated these increases and decreased the prevalence of *Toxoplasma* and the overall richness of protozoa.	Popovic et al., 2021 [77].
Kenyan infants aged 6 months (*n* = 115).	(1) MNPs containing 2.5 mg iron/day as NaFeEDTA or (2) not for 4 months and (3) MNPs containing 12.5 mg iron/day as ferrous fumarate or (4) not for 4 months.	-Lower presence of *Bifidobacterium* after ferrous fumarate MNPs administration. -Greater effect on *Escherichia*/*Shigella*, the ratio *Enterobacteria* to *Bifidobacteria* and pathogenic *E. coli* in infants receiving NaFeEDTA MNPs. -Increase in *Faecalibacterium* and *Prevotella* and a decrease in *Enterobacteriaceae*, compared to baseline, in infants receiving MNPs without Fe.	Jaeggi et al., 2015 [73]
Kenyan infants (*n* = 155) aged 6.5–9.5 months.	(1) MNPs without iron, (2) MNPs with 5 mg of iron (2.5 mg as NaFeEDTA and 2.5 mg as ferrous fumarate) or (3) the same MNPs with iron, with 7.5 g of GOS for 4 months.	-Lower concentrations of genus *Lactobacillus* and *Bifidobacterium* and greater abundances of the order *Clostridiales* and family *Enterobacteriaceae*, but no differences in phylum *Bacteroidetes*, in participants receiving iron alone. -Comparison of participants receiving iron alone vs. iron with GOS showed lower abundances of genera *Lactobacillus* and *Bifidobacterium* and higher concentrations of order *Clostridiales*, but no differences in *Enterobacteriaceae* or *Bacteroidetes*.	Paganini et al., 2017 [75]
Kenyan infants between 8 and 10 months of age (*n* = 28)	(1) Antibiotic and MNPs with 2.5 mg of iron (Ab+Fe+), (2) antibiotics and MNPs without iron (Ab+Fe), (3) no antibiotics and MNPs with 2.5 mg of iron (Ab−Fe+) or (4) no antibiotics and no iron MNPs (Ab−Fe−). Antibiotic treatment lasted 5 days whereas supplementation with MNPs, 40 days.	-Large differences in gut microbiota composition in infants receiving antibiotics with iron vs. those receiving antibiotics without iron. Decrease in abundances of *Bifidobacterium* in infants from the Ab+Fe+ group and increase in those from the Ab+Fe− subgroup. -Decrease in pathogenic *E. coli* in the Ab+Fe− subgroup. -Increase in *Clostridium difficile* in the Ab+Fe+ subgroup.	Paganini et al., 2019 [74].
Healthy Malawian infants (*n* = 160) aged 6 months	(1) 71 g/day of micronutrient-fortified corn-soy blend (5.46 mg/day of iron), (2) 54 g/day of micronutrient-fortified LONS with milk protein base (6 mg/day of iron), (3) 54 g/day of micronutrient-fortified LONS with soy protein base (6 mg/day of iron) or (4) any supplementary food for 6 months.	-No differences observed in bacterial diversity or colony counts between the intervention groups. -In the total study population, lower counts of *Bifidobacterium infantis*, *Bifidobacterium lactis* and *Bibidobacterium longum* detected, coupled with higher counts of total bacterial, *Bifidobacterium genus* and *Bifidobacterium catenulatum*. -Decrease in *Staphylococcus aureus* over time in the total study infants.	Aakko et al., 2017 [37].
Healthy Malawian infants (*n* = 160) aged 6 months	(1) No supplementary food during the primary follow-up period and 71 g/day of micronutrient-fortified corn-soy blend (5.46 mg/day of iron) for 6 months or (2) 71 g/day of micronutrient-fortified corn-soy blend (5.46 mg/day of iron), (3) 54 g/day of micronutrient-fortified LONS with milk protein base (6 mg/day of iron) or (4) 54 g/day of micronutrient-fortified LONS with soy protein base (6 mg/day of iron) for 12 months.	-No significant differences among intervention groups. -Greater counts of some *Lactobacillus* strains in participants supplemented with soy LONS. -*Bifidobacterium longus* was the most abundant species at both ages, mainly at the baseline. -Significant decrease in *Bifidobacteriaceae* and *Enterobacteriaceae* with age, whereas *Prevotella* and *Faecalibacterium* significantly increased. -No differences in *Salmonella*, *Shigella* and *Escherichia* between groups or time points.	Cheung et al., 2016 [80].
Pregnant women (*n* = 869) from Malawi.	(1) Iron and folic acid (60 mg and 400 μg, respectively); (2) 20 mg of iron, 400 μg of folic acid and 16 additional micronutrients or (3) the same micronutrients than (2) but as LONS with four additional minerals, proteins, and fat, daily during pregnancy and until 6 months postpartum. Infants from group (3) received the same supplements as mothers from 6 to 18 months and infants from groups (1) and (2) did not receive any supplements.	-No differences in microbiota diversity and maturation between groups (1) and (2). -Higher microbiota diversity in group (3) at 18 months (12 months of infant supplementation) but not in the remaining time points. -No alteration of microbiota maturation due to supplementation. -After 1 month, *Bifidobacterium* (mainly *Bifidobacterium longum*) was the most abundant genus and then gradually decrease until 30 months. -Increase of *Prevotella* abundance (predominantly *Prevotella copri*) with age, becoming one of the most abundant species at the end of the intervention. -Stabilization of *Streptococcus* abundance over time and *Faecalibacterium* increase from 6 to 30 months.	Kamng’ona et al., 2020 [81].
Gambian children with IDA aged 6–35 months (*n* = 705).	(1) Ferrous sulphate (12.5 mg of elemental iron equivalent daily), (2) IHAT (20 mg elemental iron equivalent daily, the bioequivalent dose considering the bioavailability of IHAT relative to ferrous sulphate) or (3) placebo (around 30 mg of pharmaceutical-grade sucrose daily) for 12 weeks.	-Iron supplementation did not significantly affect gut microbiota and age was main factor that determined bacterial composition of fecal samples. -Development of a *Prevotella*-rich gut microbiota during the study timeframe.	de Goffau et al., 2022 [85].
Swedish infants aged 6 months (*n* = 72)	(1) Low-iron-fortified formula (1.2 mg iron/day), (2) high iron-fortified formula (6.6 mg iron/day) or (3) no added iron formula with liquid ferrous sulfate supplementation (iron drops; 6.6 mg iron/day) for 45 days.	-Decrease in the relative abundance of *Bifidobacterium* compared with low-iron formula -No differences in pathogenic bacteria growth. -Lower relative abundance of *Lactobacillus* spp. in infants receiving iron drops vs. high-iron formula group. -Higher relative abundance of *Lactobacillus* spp. in high-iron vs. low-iron. -Lower relative abundance of *Streptococcus* in infants receiving iron drops -Greater relative abundance of *Clostridium* and *Bacteroides* in infants receiving high-iron formula.	Sjödin et al., 2019 [41].
Infants with IDA (*n* = 37) aged 9 to 24 months from Denver (Colorado)	(1) Iron supplementation (6 mg/kg/day) alone or (2) combined with vitamin E (18 mg/day) for 8 weeks.	-Decrease in *Bacteroidetes* and increase in *Firmicutes* infants receiving iron and vitamin E, relative to the iron alone group. -Decrease in genus *Escherichia* (either commensals or pathogens) among all participants.	Tang et al., 2016 [86].

IDA: iron-deficiency anemia; IHAT: iron hydroxide adipate tartrate; LONS: lipid-based nutrient supplements; MNPs: micronutrient powders; NaFeEDTA: sodium iron ethylenediaminetetra-acetate.

Since iron therapy is related to inflammatory processes that may decrease iron absorption, the combination of iron with antioxidants such as vitamin E could be an interesting approach to enhance the efficacy of iron therapy through the reduction of iron-induced inflammation. In this regard, a study carried out in infants and toddlers from the United States tested the influence of adding vitamin E to iron therapy. For this purpose, participants (*n* = 37) with IDA aged 9 to 24 months were randomly assigned to receive iron supplementation (6 mg/kg/day) alone or combined with vitamin E (18 mg/day) for 8 weeks. Primary outcomes were those related with iron status (serum ferritin, transferrin saturation, and serum transferrin receptor) and inflammation (IL-4, TNF-α, CRP, and fecal calprotectin). The gut microbiota profile was assessed before and after supplementation. Overall results suggested that vitamin E did not affect iron status. Moreover, no differences were observed in inflammatory markers (IL-4 and TNF-α) between groups. However, after intervention, fecal calprotectin concentration was higher in the group without vitamin E, although these differences did not reach statistical significance. Despite the lack of differences in primary outcomes, significant differences in microbiota composition over time were determined. Specifically, the group with iron and vitamin E showed a decreased in the relative abundance of *Bacteroidetes* and an increase in *Firmicutes*, relative to the iron alone group. These changes were mainly driven by the decrease in families *Bacteroidaceae* (phylum *Bacteroidetes*) and the increase in *Lachnospiraceae* (phylum *Firmicutes*) observed in the iron + vitamin E participants vs. those receiving iron alone. Furthermore, an increase of the genus *Roseburia*, from the phylum *Firmicutes*, was also observed in the volunteers treated with combined therapy. However, a decrease in genus *Escherichia* (either commensals or pathogens) among all participants was observed [86]. The latter results raise the possibility that the combination of iron therapy with some antioxidants (i.e., vitamin E) may potentially exert health benefits. Particularly, the relative abundance of the *Roseburia* increase leads to increases in butyrate production. Since butyrate is a preferred substrate for colonocytes and promotes gut mucosal barrier function, it may improve colonic function [87,88]. However, due to the reduced number of participants of this study, further research is needed to confirm this relevant finding.

## 4. Intervention Studies Performed in Children and Adolescents

Although the majority of the scientific evidence available to date on the effect of iron supplementation on gut microbiota comes from infants and toddlers, a few studies present data from children and adolescents (Table 2). A study performed in Bangladesh examined the effect of MNPs with standard vs. low-iron concentration on the composition of gut microbiota. Hence, children (*n* = 53) aged 2 to 5 years who drank groundwater with high iron concentration (≥2 mg/L) were randomly assigned to receive either standard MNPs sachets (with 12.5 mg of ferrous fumarate, 300 μg of vitamin A, 5 mg of Zn, 30 mg of vitamin C and 0.15 mg of folic acid) or low-iron MNPs sachets (with the same composition except for 5 mg of ferrous fumarate). The intervention lasted for two months, and each participant received one sachet per day. Serum ferritin and CRP, hemoglobin, and AGP were selected as outcomes, in addition to the analysis of gut microbiome in fecal samples. No differences in microbiota composition at the endpoint were observed between the groups. Since at baseline, some young children had a relatively adult-like microbiota and some older children a younger microbiota, the microbiome age of each child was determined in order to categorize children as having a young or relatively old microbiota. Thus, in the old-microbiome subgroup, a higher relative abundance of certain beneficial species (*Bifidobacterium* and *Lactobacillus*) as well as the potentially pathogenic *Enterobacteriaceae* were found in the group of standard MNPs, compared with the low iron group (without significant differences among groups). The higher relative abundance of *Bifidobacterium* and *Lactobacillus* was unexpected, as iron tends to decrease the normal growth of these beneficial bacteria. The authors explained this finding, considering that at the end of the study, the intestinal iron load was increased by MNPs consumption in both groups. As a result, the combination of high iron load in the gut and lower relative abundance of *Bifidobacterium* and *Lactobacillus* at baseline was mitigated at the endpoint [89].

Zimmermann et al. [90] evaluated the effect of iron fortification on gut microbiota and gut inflammation in children and adolescents from Côte d´Ivoire. This double-blind controlled trial was conducted amongst children aged 6 to 14 years (*n* = 139) for six months who received iron-fortified biscuits with 20 mg of electrolytic iron four times per week or non-fortified biscuits. At baseline and after 6 months of intervention, blood and stool samples were assessed. Blood analysis encompassed hemoglobin, zinc protoporphyrin, serum ferritin, and CRP concentrations. The fecal analysis comprised analysis of gut microbiome by PCR, detection of gut pathogens and determination of calprotectin concentration. Iron fortification was ineffective, and no differences were observed in iron serum status at the end of the intervention. However, iron fortification modified the gut microbiota. Specifically, compared with the control group, an increase in enterobacteria concentration (*Salmonella* spp., *Shigella* spp. and/or *E. coli*) and a decrease in *Lactobacillus* population was observed. In addition, a high concentration of fecal calprotectin was detected, suggesting that changes in gut microbiota may induce gut inflammation [90]. These results could be attributed to the fact that, as previously stated, for most enteric Gram-negative bacteria, such as *Salmonella*, *Shigella* or pathogenic *E. coli*, iron plays a crucial role being essential for their growth and virulence, whereas beneficial intestinal barrier bacteria, such as *Lactobacillus* do not require iron for survival [46]. Therefore, increased levels of iron in the gut may have unfavorable effects for the host.

At a later date, the same authors carried out another study on children (*n* = 73) aged 6 to 11 years old from two different schools from South Africa. Iron-deficient children were randomly divided into two different subgroups: one received iron supplements (one tablet of 50 mg of FeSO_4_/day for 4 weeks; *n* = 22), whereas the other received a placebo (*n* = 27). Stool samples were analyzed at baseline and after 2, 12 and 38 weeks of supplementation, including selected bacterial target groups in the gut (*Bacteroides* ssp., *Firmicutes*, *Enterobacteriacea*, and *Lactobacillus* ssp., among others), fecal SCFA concentration and fecal calprotectin concentration (indicator of gut inflammation). The main outcomes revealed that iron supplementation did not significantly modify the concentrations of the dominant gut bacteria nor SCFA and calprotectin concentrations compared with the placebo group. Furthermore, the prevalent bacteria in the gut and fecal SCFA concentrations in iron-deficient and iron-sufficient children were similar. These findings may be justified by the impact of environmental factors (such as, for example, poor-quality water or food supplies with increased exposure to enteropathogens) upon the effect of supplementation on gut microbiota [91].

## 5. Intervention Studies Performed in Adults

The summary of the effects of the intervention studies performed in adults on microbiota are shown in Table 3. Since ID and IDA are frequent complications in colorectal cancer, therefore, iron supplementation is often needed. In addition, alterations in the gut bacterial ecosystem are characteristic of this type of cancer [92] and alongside this, orally administered drugs may alter gut microbiota [93]. As previously stated, iron supplementation has the potential to modify bacterial populations of the colorectal tumor-associated (procarcinogenic or on-tumor) gut microbiota as well as the non-tumor-associated (off-tumor) microbiota, by means of an increase of iron concentration in gut lumen [94]. To avoid this matter, it has been suggested that iron intravenous administration could be more beneficial in colorectal cancer in order to limit microbial perturbations associated with oral iron [95]. Therefore, detailed intervention research studies based on the effects of the interventions performed in adults on microbiota have been carried out (Table 3). Specifically, a study was conducted to confirm that intravenous administration of iron might be more beneficial in non-metastatic colorectal cancer in anemic patients (*n* = 40). Thus, patients were randomized to receive twice-daily oral ferrous sulfate (200 mg) or intravenous iron as ferric carboxymaltose (dosed by body weight and hemoglobin concentration according to the product characteristics). The intervention lasted an average of 26.5 days in case of oral iron and 23.5 days in case of the intravenous iron. The microbiota was characterized from colorectal tumor biopsies. When analyzing if the administration method changed on- and off-tumor microbiota, it was observed that patients who received oral iron showed an off-tumor microbiota enriched with *Bacteroidaceae* family and *Bacteroides* genus while the on-tumor microbiota showed a greater abundance of *Nocardiaceae*, *Intrasporangiaceae*, and *Brevibacteriaceae* families and *Prevotella*, *Nocardioides*, *Kocuria*, *Brevibacterium*, *Veillonella* and *Catenibacterium* genus. In contrast, patients treated with intravenous iron showed an enrichment of their off-tumor microbiota with the *Firmicutes* phylum and the *Clostridia* spp. along with higher abundances of the Clostridiales and Sphingomonadales orders, the *Sphingomonadaceae* family and the *Paraprevotella* genus. Regarding the on-tumor microbiota of these patients, a higher abundance of *Epsilonbacteraeota* phylum, Campylobacteria class, Campylobacteriales order, *Campylobacteraceae*, *Propionibacteriaceae* and *Porphyromonadaceae* families and *Campylobacter*, *Porphyromonas* and *Cutibacterium* were observed. These complex results indicate that patients treated with oral iron had a more consistent tumor-associated and tumor-adjacent microbiota, whereas those who received intravenous iron showed greater differences between their tumor-associated and tumor adjacent microbiota, that is between the tumor and non-tumor colonic tissue. Moreover, comparison of predictive metagenomics between iron treatment groups showed that oral iron-treated patients have potentially more procarcinogenic on- and off-tumor microbiota compared to intravenous iron-treated patients. This infers that intravenous iron seems to be more beneficial for the management of severe anemia in colorectal cancer patients, in order to avoid gut microbiota disturbances associated with oral iron supplements [96].

Owing to the different problems related to oral iron absorption, diverse strategies have been studied in the last few years, including its administration as iron fumarate, gluconate, carbonyl iron, and polysaccharide-iron complex [97]. Recently, lactoferrin, a glycoprotein extracted from milk or whey, has been proposed as a supplement to enhance immunity, support digestive health, and increase iron absorption, among others [98]. The main drawback related to lactoferrin use is that when orally consumed, it may be lost through digestion in the stomach and thus do not reach the gut in sufficient amounts. To avoid this issue, a novel encapsulated lactoferrin has been commercialized, the so-called Inferrin^TM^ based on alginate microgels. This novel formulation protects lactoferrin in low pH conditions such as the stomach, but releases it in neutral pH conditions, such as the gut [99]. In order to compare the absorption and efficacy of Inferrin^TM^ with conventional lactoferrin and their impact on gut microbiota, a double-blind randomized crossover trial was conducted with 12 healthy males. Each therapeutic arm lasted four weeks with a two-week washout period in between. Two doses of lactoferrin were tested (200 or 600 mg), either as convention formulation or as Inferrin^TM^. Blood tests were performed at baseline, in the middle and at the end of supplementation with each component and fecal samples were collected at the baseline and at the end of each trial period. Fecal microbiome was characterized using 16S and metabolomic sequencing. An increased amount of lactoferrin was found in stool samples in the case of Inferrin^TM^ at higher doses (600 mg), suggesting that the lactoferrin encapsulation was effective, and protects it against gastric degradation, although it may not be fully absorbed. Concerning gut microbiome, it appears that lactoferrin decreased the levels of *Euryarchaeota*, *Acidobacterial*, *Chloroflexi*, NC10, and *Nitrospirae* and increased the levels of *Firmicutes* and *Bacteroidetes*. These results were especially significant when the patients were supplemented with 600 mg of lactoferrin, regardless of encapsulation. These results suggested that lactoferrin may have beneficial effects on the microbiome, although larger detailed studies are needed [100].

Inflammatory bowel disease is a result of disordered immune responses to commensal and pathogenic gut microbes in genetically susceptible individuals [101]. IDA is frequently observed in patients with inflammatory bowel disease, which often need iron supplementation. Studies that evaluated the most appropriate route of iron administration in these patients have led to contradictory results [102,103]. Therefore, Lee et al. [104] compared the effects of oral and intravenous iron therapy in ID patients from Canada (*n* = 72). Specifically, the patients with Crohn´s disease (*n* = 31), ulcerative colitis (*n* = 22) or the controls (*n* = 19) were randomly allocated to receive either oral (300 mg, twice a day) or intravenous (three or four separate iron sucrose 300 mg infusions if ID only or with anemia, respectively) for 12 weeks. Clinical parameters, fecal bacterial communities and metabolomes were analyzed before and after intervention. The route of administration did not affect the hemoglobin levels. However, the iron content in feces was significantly increased after oral iron administration, but not after intravenous treatment. Regarding gut microbiota, lower abundances of *Collinsella aerofaciens*, *Faecalibacterium prausnitzii*, *Ruminococcus bromii* and *Dorea* spp. and higher abundances of the genus *Bifidobacterium* were observed after oral iron therapy. The iron route of administration did not lead to changes in disease activity. After iron administration, major shifts in bacterial diversity were observed in approximately half of the participants, but the bacterial communities of patients with Crohn´s disease were the most susceptible. The obtained results confirmed that oral administration differentially affects bacterial phylotypes and fecal metabolites compared with intravenous therapy. Moreover, intravenous iron therapy may be potentially interesting for anemic patients with CD with an instable microbiota, considering the greater sensibility of their microbiota to changes associated with oral iron administration [104]. 

## 6. Conclusions

Overall, the available evidence suggests that iron supplementation should be performed with caution due to the possible alterations it may cause amongst the gut microbiota. Regardless, large population studies are still required to clarify the somewhat contradictory results. Likewise, supplementation with iron should not be done indiscriminately but should be targeted to those individuals with iron deficiency or iron-deficiency anemia. On the other hand, the lack of studies on the elderly population, often supplemented with iron, is surprising. In view of the above, physicians, pharmacists, nurses, and dietitian-nutritionists coordinate their efforts to improve public awareness of the rational use of iron supplementation.

## Figures and Tables

**Table 2:** Summary of the effects of the intervention studies performed in children and adolescents on gut microbiota.

Study Subjects	Intervention	Effects on Gut Microbiota	Author and Year
Children (*n* = 53) from Bangladesh aged 2 to 5 years.	(1) MNPs sachets (with 12.5 mg of ferrous fumarate, 300 μg of vitamin A, 5 mg of Zn, 30 mg of vitamin C and 0.15 mg of folic acid) or (2) a low iron MNPs sachets (with the same composition except for 5 mg of ferrous fumarate) for 2 months.	-No differences in microbiota composition at the endpoint between groups. -Higher relative abundance of certain beneficial bacteria, *Bifidobacterium* and *Lactobacillus*, and other pathogenic, *Enterobacteriaceae*, in infants receiving standard MNPs, compared with the low iron group.	Rahman et al., 2021 [89].
Children and adolescents from Côte d´Ivoire aged 6 to 14 years (*n* = 139).	(1) Iron-fortified biscuits with 20 mg of electrolytic iron 4 times per week or (2) non-fortified biscuits for 6-months.	-Increase in enterobacteria concentration (*Salmonella* spp., *Shigella* spp and/or *E. coli*) and decrease in *Lactobacillus* population, compared with the control group.	Zimmermann et al., 2010 [90].
Iron-deficient children (*n* = 73) aged 6 to 11 years old from South Africa.	(1) One tablet of 50 mg of FeSO_4_/day for 4 weeks or (2) placebo.	-Iron supplementation did not significantly modify the concentrations of the dominant gut bacteria, compared with the placebo group.	Dostal et al., 2014 [91].

MNPs: micronutrient powders.

**Table 3:** Summary of the effects of the intervention studies performed in adults on microbiota.

Study Subjects	Intervention	Effects on Gut Microbiota	Author and Year
Anemic patients (*n* = 40) with non-metastatic colorectal cancer.	(1) Oral ferrous sulfate twice a day (200 mg), (2) or intravenous iron as ferric carboxymaltose (dosed by body weight and hemoglobin concentration according to the product characteristics) for 26.5 days and 23.5 days, respectively.	-Off-tumor microbiota enriched with *Bacteroidaceae* family and *Bacteroides* genus while the on-tumor microbiota showed a greater abundance of *Nocardiaceae*, *Intrasporangiaceae*, and *Brevibacteriaceae* families and *Prevotella*, *Nocardioides*, *Kocuria*, *Brevibacterium*, *Veillonella* and *Catenibacterium* genus in patients receiving oral iron. -Enrichment of off-tumor microbiota with the *Firmicutes* phylum and the *Clostridia* spp. along with higher abundances of the Clostridiales and Sphingomonadales orders, the *Sphingomonadaceae* family and the *Paraprevotella* genus in patients receiving intravenous iron. -Moreover, the on-tumor microbiota with higher abundance of Epsilonbacteraeota phylum, Campylobacteria class, Campylobacteriales order, *Campylobacteraceae*, *Propionibacteriaceae* and *Porphyromonadaceae* families and *Campylobacter*, *Porphyromonas* and *Cutibacterium*.	Phipps et al., 2021 [96].
Healthy males (*n* = 12).	(1) 200, or (2) 600 mg of lactoferrin as convention formulation, or (3) 200, or (4) 600 mg of lactoferrin as Inferrin^TM^ for 4 weeks.	-Decreased levels of *Euryarchaeota*, *Acidobacterial*, *Chloroflexi*, NC10, and *Nitrospirae* and increased levels of *Firmicutes* and *Bacteroidetes*, especially in patients supplemented with 600 mg of lactoferrin, regardless of encapsulation.	Dix et al., 2018 [100].
Iron-deficient patients from Canada (*n* = 72) with Crohn´s disease, ulcerative colitis or controls	(1) Oral (300 mg, twice a day), or (2) intravenous (three or four separate iron sucrose 300 mg infusions if ID only or with anemia, respectively) for 12 weeks.	-Lower abundances of *Collinsella aerofaciens*, *Faecalibacterium prausnitzii*, *Ruminococcus bromii* and *Dorea* sp. and higher abundances of the genus *Bifidobacterium* after oral iron therapy. -Major shifts in bacterial diversity after iron administration in approximately half of the participants, with the bacterial communities of patients with Crohn’s disease being the most susceptible.	Lee et al., 2017 [104].

## Data Availability

Not applicable.

## References

[1] Albenberg L.G., Wu G.D. (2014). Diet and the Intestinal Microbiome: Associations, Functions, and Implications for Health and Disease. Gastroenterology.

[2] Sekirov I., Russell S.L., Antunes L.C.M., Finlay B.B. (2010). Gut Microbiota in Health and Disease. Physiol. Rev..

[3] Bolte L.A., Vich Vila A., Imhann F., Collij V., Gacesa R., Peters V., Wijmenga C., Kurilshikov A., Campmans-Kuijpers M.J.E., Fu J. (2021). Long-term dietary patterns are associated with pro-inflammatory and anti-inflammatory features of the gut microbiome. Gut.

[4] Thursby E., Juge N. (2017). Introduction to the human gut microbiota. Biochem. J..

[5] Qin J., Li R., Raes J., Arumugam M., Burgdorf K.S., Manichanh C., Nielsen T., Pons N., Levenez F., Yamada T. (2010). A human gut microbial gene catalogue established by metagenomic sequencing. Nature.

[6] Eckburg P.B., Bik E.M., Bernstein C.N., Purdom E., Dethlefsen L., Sargent M., Gill S.R., Nelson K.E., Relman D.A. (2005). Diversity of the Human Intestinal Microbial Flora. Science.

[7] Bäckhed F., Ley R.E., Sonnenburg J.L., Peterson D.A., Gordon J.I. (2005). Host-Bacterial Mutualism in the Human Intestine. Science.

[8] Maslowski K.M., Mackay C.R. (2010). Diet, gut microbiota and immune responses. Nat. Immunol..

[9] Matamoros S., Guen C.G.-L., Le Vacon F., Potel G., de La Cochetiere M.-F. (2013). Development of intestinal microbiota in infants and its impact on health. Trends Microbiol..

[10] Lyons K.E., Ryan C.A., Dempsey E.M., Ross R.P., Stanton C. (2020). Breast Milk, a Source of Beneficial Microbes and Associated Benefits for Infant Health. Nutrients.

[11] Fabiano V., Indrio F., Verduci E., Calcaterra V., Pop T.L., Mari A., Zuccotti G.V., Cokugras F.C., Pettoello-Mantovani M., Goulet O. (2021). Term Infant Formulas Influencing Gut Microbiota: An Overview. Nutrients.

[12] Rinninella E., Raoul P., Cintoni M., Franceschi F., Miggiano G.A.D., Gasbarrini A., Mele M.C. (2019). What Is the Healthy Gut Microbiota Composition? A Changing Ecosystem across Age, Environment, Diet, and Diseases. Microorganisms.

[13] Bull M.J., Plummer N.T. (2014). Part 1: The Human Gut Microbiome in Health and Disease. Integr. Med..

[14] O’Keefe S.J.D. (2008). Nutrition and colonic health: The critical role of the microbiota. Curr. Opin. Gastroenterol..

[15] Fan Y., Pedersen O. (2021). Gut microbiota in human metabolic health and disease. Nat. Rev. Microbiol..

[16] De Filippo C., Cavalieri D., Di Paola M., Ramazzotti M., Poullet J.B., Massart S., Collini S., Pieraccini G., Lionetti P. (2010). Impact of diet in shaping gut microbiota revealed by a comparative study in children from Europe and rural Africa. Proc. Natl. Acad. Sci. USA.

[17] Yatsunenko T., Rey F.E., Manary M.J., Trehan I., Dominguez-Bello M.G., Contreras M., Magris M., Hidalgo G., Baldassano R.N., Anokhin A.P. (2012). Human gut microbiome viewed across age and geography. Nature.

[18] Kortekangas E., Kamng’Ona A.W., Fan Y., Cheung Y.B., Ashorn U., Matchado A., Poelman B., Maleta K., Dewey K.G., Ashorn P. (2020). Environmental exposures and child and maternal gut microbiota in rural Malawi. Paediatr. Périnat. Epidemiol..

[19] Tomova A., Bukovsky I., Rembert E., Yonas W., Alwarith J., Barnard N.D., Kahleova H. (2019). The Effects of Vegetarian and Vegan Diets on Gut Microbiota. Front. Nutr..

[20] De Filippis F., Pellegrini N., Vannini L., Jeffery I.B., La Storia A., Laghi L., Serrazanetti D.I., Di Cagno R., Ferrocino I., Lazzi C. (2016). High-level adherence to a Mediterranean diet beneficially impacts the gut microbiota and associated metabolome. Gut Microbiota.

[21] Merra G., Noce A., Marrone G., Cintoni M., Tarsitano M.G., Capacci A., De Lorenzo A. (2021). Influence of Mediterranean Diet on Human Gut Microbiota. Nutrients.

[22] Desai M.S., Seekatz A.M., Koropatkin N.M., Kamada N., Hickey C.A., Wolter M., Pudlo N.A., Kitamoto S., Terrapon N., Muller A. (2016). A Dietary Fiber-Deprived Gut Microbiota Degrades the Colonic Mucus Barrier and Enhances Pathogen Susceptibility. Cell.

[23] Slavin J. (2003). Why whole grains are protective: Biological mechanisms. Proc. Nutr. Soc..

[24] Levine A., Boneh R.S., Wine E. (2018). Evolving role of diet in the pathogenesis and treatment of inflammatory bowel diseases. Gut.

[25] Forbes J.D., Chen C.-Y., Knox N.C., Marrie R.-A., El-Gabalawy H., De Kievit T., Alfa M., Bernstein C.N., Van Domselaar G. (2018). A comparative study of the gut microbiota in immune-mediated inflammatory diseases—Does a common dysbiosis exist?. Microbiome.

[26] Kalinkovich A., Livshits G. (2019). A cross talk between dysbiosis and gut-associated immune system governs the development of inflammatory arthropathies. Semin. Arthritis Rheum..

[27] Zitvogel L., Pietrocola F., Kroemer G. (2017). Nutrition, inflammation and cancer. Nat. Immunol..

[28] Erridge C. (2011). Diet, commensals and the intestine as sources of pathogen-associated molecular patterns in atherosclerosis, type 2 diabetes and non-alcoholic fatty liver disease. Atherosclerosis.

[29] Arrieta M.-C., Arévalo A., Stiemsma L., Dimitriu P., Chico M.E., Loor S., Vaca M., Boutin R.C., Morien E., Jin M. (2017). Associations between infant fungal and bacterial dysbiosis and childhood atopic wheeze in a nonindustrialized setting. J. Allergy Clin. Immunol..

[30] Vatanen T., Franzosa E.A., Schwager R., Tripathi S., Arthur T.D., Vehik K., Lernmark Å., Hagopian W.A., Rewers M.J., She J.-X. (2018). The human gut microbiome in early-onset type 1 diabetes from the TEDDY study. Nature.

[31] Abbaspour N., Hurrell R., Kelishadi R. (2014). Review on iron and its importance for human health. J. Res. Med. Sci. Off. J. Isfahan Univ. Med. Sci..

[32] Haas J.D., Brownlie IV T. (2001). Iron deficiency and reduced work capacity: A critical review of the research to determine a causal relationship. J. Nutr..

[33] World Health Organization (2018). Weekly Iron and Folic Acid Supplementation as an Anaemia-Prevention Strategy in Women and Adolescent Girls: Lessons Learnt from Implementation of Programmes among Non-Pregnant Women of Reproductive Age.

[34] Milman N., Taylor C.L., Merkel J., Brannon P.M. (2017). Iron status in pregnant women and women of reproductive age in Europe. Am. J. Clin. Nutr..

[35] Hercberg S., Preziosi P., Galan P. (2001). Iron deficiency in Europe. Public Health Nutr..

[36] EFSA Panel on Dietetic Products, Nutrition and Allergies (NDA) (2015). Scientific opinion on dietary reference values for iron. EFSA J..

[37] Aakko J., Grzeskowiak L., Asukas T., Päivänsäde E., Lehto K.-M., Fan Y.-M., Mangani C., Maleta K., Ashorn P., Salminen S. (2017). Lipid-based Nutrient Supplements Do Not Affect Gut Bifidobacterium Microbiota in Malawian Infants: A Randomized Trial. J. Pediatr. Gastroenterol. Nutr..

[38] De Lourdes Samaniego-Vaesken M., Partearroyo T., Olza J., Aranceta-Bartrina J., Gil Á., González-Gross M., Ortega R.M., Serra-Majem L., Varela-Moreiras G. (2017). Iron Intake and Dietary Sources in the Spanish Population: Findings from the ANIBES Study. Nutrients.

[39] Rahman M., Abe S.K., Rahman S., Kanda M., Narita S., Bilano V., Ota E., Gilmour S., Shibuya K. (2016). Maternal anemia and risk of adverse birth and health outcomes in low- and middle-income countries: Systematic review and meta-analysis1,2. Am. J. Clin. Nutr..

[40] Institute of Medicine (US) Panel on Micronutrients (2001). Dietary Reference Intakes for Vitamin A, Vitamin K, Arsenic, Boron, Chromium, Copper, Iodine, Iron, Manganese, Molybdenum, Nickel, Silicon, Vanadium, and Zinc.

[41] Sjödin K.S., Domellöf M., Lagerqvist C., Hernell O., Lönnerdal B., A Szymlek-Gay E., Sjödin A., E West C., Lind T. (2018). Administration of ferrous sulfate drops has significant effects on the gut microbiota of iron-sufficient infants: A randomised controlled study. Gut.

[42] World Health Organization (2012). Guideline: Daily Iron and Folic Acid Supplementation in Pregnant Women.

[43] Angoro B., Motshakeri M., Hemmaway C., Svirskis D., Sharma M. (2022). Non-transferrin bound iron. Clin. Chim. Acta.

[44] Paganini D., Zimmermann M.B. (2017). The effects of iron fortification and supplementation on the gut microbiome and diarrhea in infants and children: A review. Am. J. Clin. Nutr..

[45] Naikare H., Palyada K., Panciera R., Marlow D., Stintzi A. (2006). Major Role for FeoB in *Campylobacter jejuni* Ferrous Iron Acquisition, Gut Colonization, and Intracellular Survival. Infect. Immun..

[46] Krebs N.F., Domellöf M., Ziegler E. (2015). Balancing Benefits and Risks of Iron Fortification in Resource-Rich Countries. J. Pediatr..

[47] Lievin V., Peiffer I., Hudault S., Rochat F., Brassart D., Neeser J.-R., Servin A.L. (2000). Bifidobacterium strains from resident infant human gastrointestinal microflora exert antimicrobial activity. Gut.

[48] Goheen M.M., Bah A., Wegmüller R., Verhoef H., Darboe B., Danso E., Prentice A.M., Cerami C. (2017). Host iron status and erythropoietic response to iron supplementation determines susceptibility to the RBC stage of falciparum malaria during pregnancy. Sci. Rep..

[49] UNICEF, WHO, World Bank Group (2021). Levels and Trends in Child Malnutrition: UNICEF/WHO/The world Bank Group Joint Child Malnutrition Estimates: Key Findings of the 2021 Edition.

[50] Black R.e., Victora C.g., Walker S.P., Bhutta Z.A., Christian P., de Onis M., Ezzati M., Grantham-McGregor S., Katz J., Martorell R. (2013). Maternal and child undernutrition and overweight in low-income and middle-income countries. Lancet.

[51] Liu L., Oza S., Hogan D., Chu Y., Perin J., Zhu J., Lawn J.E., Cousens S., Mathers C., Black R.E. (2016). Global, regional, and national causes of under-5 mortality in 2000–2015: An updated systematic analysis with implications for the Sustainable Development Goals. Lancet.

[52] Subramanian S., Huq S., Yatsunenko T., Haque R., Mahfuz M., Alam M.A., Benezra A., DeStefano J., Meier M.F., Muegge B. (2014). Persistent gut microbiota immaturity in malnourished Bangladeshi children. Nature.

[53] Smith M.I., Yatsunenko T., Manary M.J., Trehan I., Mkakosya R., Cheng J., Kau A.L., Rich S.S., Concannon P., Mychaleckyj J.C. (2013). Gut Microbiomes of Malawian Twin Pairs Discordant for Kwashiorkor. Science.

[54] Kortman G.A., Raffatellu M., Swinkels D.W., Tjalsma H. (2014). Nutritional iron turned inside out: Intestinal stress from a gut microbial perspective. FEMS Microbiol. Rev..

[55] Mayo-Wilson E., Junior J., Imdad A., Dean S., Chan X.H., Chan E.S., Jaswal A., A Bhutta Z. (2014). Zinc supplementation for preventing mortality, morbidity, and growth failure in children aged 6 months to 12 years of age. Cochrane Database Syst. Rev..

[56] Imdad A., Herzer K., Mayo-Wilson E., Yakoob M.Y., Bhutta Z.A. (2010). Vitamin A supplementation for preventing morbidity and mortality in children from 6 months to 5 years of age. Cochrane Database Syst. Rev..

[57] WHO/UNICEF/UNU (2001). Iron Deficiency anaemia: Assessment, Prevention and Control: A Guide for Programme Managers.

[58] Gera T. (2002). Effect of iron supplementation on incidence of infectious illness in children: Systematic review. BMJ.

[59] Iannotti L.L., Tielsch J.M., Black M.M., Black R.E. (2006). Iron supplementation in early childhood: Health benefits and risks. Am. J. Clin. Nutr..

[60] Zimmermann M.B., Hurrell R.F. (2007). Nutritional iron deficiency. Lancet.

[61] Muriuki J.M., Mentzer A.J., Mitchell R., Webb E.L., Etyang A.O., Kyobutungi C., Morovat A., Kimita W., Ndungu F.M., Macharia A.W. (2021). Malaria is a cause of iron deficiency in African children. Nat. Med..

[62] Sazawal S., Black R.E., Ramsan M., Chwaya H.M., Stoltzfus R.J., Dutta A., Dhingra U., Kabole I., Deb S., Othman M.K. (2006). Effects of routine prophylactic supplementation with iron and folic acid on admission to hospital and mortality in preschool children in a high malaria transmission setting: Community-based, randomised, placebo-controlled trial. Lancet.

[63] Glinz D., Hurrell R.F., A Righetti A., Zeder C., Adiossan L.G., Tjalsma H., Utzinger J., Zimmermann M.B., N’Goran E.K., Wegmüller R. (2015). In Ivorian school-age children, infection with hookworm does not reduce dietary iron absorption or systemic iron utilization, whereas afebrile Plasmodium falciparum infection reduces iron absorption by half. Am. J. Clin. Nutr..

[64] Nemeth E., Tuttle M.S., Powelson J., Vaughn M.B., Donovan A., Ward D.M.V., Ganz T., Kaplan J. (2004). Hepcidin Regulates Cellular Iron Efflux by Binding to Ferroportin and Inducing Its Internalization. Science.

[65] Jonker F.A.M., Calis J.C.J., Van Hensbroek M.B., Phiri K., Geskus R.B., Brabin B., Leenstra T. (2012). Iron Status Predicts Malaria Risk in Malawian Preschool Children. PLoS ONE.

[66] Nyakeriga A.M., Troye-Blomberg M., Chemtai A.K., Marsh K., Williams T.N. (2004). Malaria and nutritional status in children living on the coast of Kenya. Am. J. Clin. Nutr..

[67] Mevissen-Verhage E.A.E., Marcelis J.H., Amerongen W.C.M.H.-V., de Vos N.M., Berkel J., Verhoef J. (1985). Effect of iron on neonatal gut flora during the first week of life. Eur. J. Clin. Microbiol..

[68] Mevissen-Verhage E.A.E., Marcelis J.H., Amerongen W.C.M.H.-V., De Vos N.M., Verhoef J. (1985). Effect of iron on neonatal gut flora during the first three months of life. Eur. J. Clin. Microbiol..

[69] Owolabi A., Senbanjo I., Oshikoya K., Boekhorst J., Eijlander R., Kortman G., Hageman J., Samuel F., Melse-Boonstra A., Schaafsma A. (2021). Multi-Nutrient Fortified Dairy-Based Drink Reduces Anaemia without Observed Adverse Effects on Gut Microbiota in Anaemic Malnourished Nigerian Toddlers: A Randomised Dose–Response Study. Nutrients.

[70] Miniello V.L., Verga M.C., Miniello A., Di Mauro C., Diaferio L., Francavilla R. (2021). Complementary Feeding and Iron Status: “*The Unbearable Lightness of Being*” Infants. Nutrients.

[71] Krebs N.F., Sherlock L.G., Westcott J., Culbertson D., Hambidge K.M., Feazel L.M., Robertson C., Frank D.N. (2013). Effects of Different Complementary Feeding Regimens on Iron Status and Enteric Microbiota in Breastfed Infants. J. Pediatr..

[72] Tang M., Frank D.N., Hendricks A.E., Ir D., Esamai F., Liechty E., Hambidge K.M., Krebs N.F. (2017). Iron in Micronutrient Powder Promotes an Unfavorable Gut Microbiota in Kenyan Infants. Nutrients.

[73] Jaeggi T., Kortman G.A.M., Moretti D., Chassard C., Holding P., Dostal A., Boekhorst J., Timmerman H.M., Swinkels D.W., Tjalsma H. (2014). Iron fortification adversely affects the gut microbiome, increases pathogen abundance and induces intestinal inflammation in Kenyan infants. Gut.

[74] Paganini D., A Uyoga M., Kortman G.A.M., I Cercamondi C., Winkler H., Boekhorst J., Moretti D., Lacroix C., Karanja S., Zimmermann M.B. (2018). Iron-containing micronutrient powders modify the effect of oral antibiotics on the infant gut microbiome and increase post-antibiotic diarrhoea risk: A controlled study in Kenya. Gut.

[75] Paganini D., A Uyoga M., Kortman G.A.M., Cercamondi C.I., Moretti D., Barth-Jaeggi T., Schwab C., Boekhorst J., Timmerman H.M., Lacroix C. (2017). Prebiotic galacto-oligosaccharides mitigate the adverse effects of iron fortification on the gut microbiome: A randomised controlled study in Kenyan infants. Gut.

[76] Soofi S., Cousens S., Iqbal S.P., Akhund T., Khan J., Ahmed I., Zaidi A.K., Bhutta Z.A. (2013). Effect of provision of daily zinc and iron with several micronutrients on growth and morbidity among young children in Pakistan: A cluster-randomised trial. Lancet.

[77] Popovic A., Bourdon C., Wang P.W., Guttman D.S., Soofi S., Bhutta Z.A., Bandsma R.H.J., Parkinson J., Pell L.G. (2021). Micronutrient supplements can promote disruptive protozoan and fungal communities in the developing infant gut. Nat. Commun..

[78] Almeida R.S., Wilson D., Hube B. (2009). *Candida albicans* iron acquisition within the host. FEMS Yeast Res..

[79] Gera T., Pena-Rosas J.P., Boy-Mena E., Sachdev H.S. (2017). Lipid based nutrient supplements (LONS) for treatment of children (6 months to 59 months) with moderate acute malnutrition (MAM): A systematic review. PLoS ONE.

[80] Cheung Y.B., Xu Y., Mangani C., Fan Y.-M., Dewey K.G., Salminen S.J., Maleta K., Ashorn P., Maleta K. (2015). Gut microbiota in Malawian infants in a nutritional supplementation trial. Trop. Med. Int. Health.

[81] Kamng’Ona A.W., Young R., Arnold C.D., Patson N., Jorgensen J.M., Kortekangas E., Chaima D., Malamba C., Ashorn U., Cheung Y.B. (2020). Provision of Lipid-Based Nutrient Supplements to Mothers During Pregnancy and 6 Months Postpartum and to Their Infants from 6 to 18 Months Promotes Infant Gut Microbiota Diversity at 18 Months of Age but Not Microbiota Maturation in a Rural Malawian Setting: Secondary Outcomes of a Randomized Trial. J. Nutr..

[82] Powell J.J., Bruggraber S.F., Faria N., Poots L.K., Hondow N., Pennycook T., Latunde-Dada G.O., Simpson R., Brown A., Pereira D. (2014). A nano-disperse ferritin-core mimetic that efficiently corrects anemia without luminal iron redox activity. Nanomed. Nanotechnol. Biol. Med..

[83] Pereira D.I.A., Bruggraber S.F.A., Faria N., Poots L.K., Tagmount M.A., Aslam M.F., Frazer D.M., Vulpe C.D., Anderson G.J., Powell J.J. (2014). Nanoparticulate iron(III) oxo-hydroxide delivers safe iron that is well absorbed and utilised in humans. Nanomed. Nanotechnol. Biol. Med..

[84] Pereira D.I., Mohammed N.I., Ofordile O., Camara F., Baldeh B., Mendy T., Sanyang C., Jallow A.T., Hossain I., Wason J. (2018). A novel nano-iron supplement to safely combat iron deficiency and anaemia in young children: The IHAT-GUT double-blind, randomised, placebo-controlled trial protocol. Gates Open Res..

[85] De Goffau M.C., Jallow A.T., Sanyang C., Prentice A.M., Meagher N., Price D.J., Revill P.A., Parkhill J., Pereira D.I.A., Wagner J. (2021). Gut microbiomes from Gambian infants reveal the development of a non-industrialized Prevotella-based trophic network. Nat. Microbiol..

[86] Tang M., Frank D.N., Sherlock L., Ir D., Robertson C.E., Krebs N.F. (2016). Effect of Vitamin E With Therapeutic Iron Supplementation on Iron Repletion and Gut Microbiome in US Iron Deficient Infants and Toddlers. J. Pediatr. Gastroenterol. Nutr..

[87] Kelly C.J., Zheng L., Campbell E.L., Saeedi B., Scholz C.C., Bayless A.J., Wilson K.E., Glover L.E., Kominsky D.J., Magnuson A. (2015). Crosstalk between Microbiota-Derived Short-Chain Fatty Acids and Intestinal Epithelial HIF Augments Tissue Barrier Function. Cell Host Microbe.

[88] Singh N., Gurav A., Sivaprakasam S., Brady E., Padia R., Shi H., Thangaraju M., Prasad P.D., Manicassamy S., Munn D.H. (2014). Activation of Gpr109a, Receptor for Niacin and the Commensal Metabolite Butyrate, Suppresses Colonic Inflammation and Carcinogenesis. Immunity.

[89] Rahman S., Kortman G.A.M., Boekhorst J., Lee P., Khan M.R., Ahmed F. (2021). Effect of low-iron micronutrient powder (MNP) on the composition of gut microbiota of Bangladeshi children in a high-iron groundwater setting: A randomized controlled trial. Eur. J. Nutr..

[90] Zimmermann M.B., Chassard C., Rohner F., N’Goran E.K., Nindjin C., Dostal A., Utzinger J., Ghattas H., Lacroix C., Hurrell R.F. (2010). The effects of iron fortification on the gut microbiota in African children: A randomized controlled trial in Côte d’Ivoire. Am. J. Clin. Nutr..

[91] Dostal A., Baumgartner J., Riesen N., Chassard C., Smuts C., Zimmermann M.B., Lacroix C. (2014). Effects of iron supplementation on dominant bacterial groups in the gut, faecal SCFA and gut inflammation: A randomised, placebo-controlled intervention trial in South African children. Br. J. Nutr..

[92] Sánchez-Alcoholado L., Ramos-Molina B., Otero A., Laborda-Illanes A., Ordóñez R., Medina J.A., Gómez-Millán J., Queipo-Ortuño M.I. (2020). The Role of the Gut Microbiome in Colorectal Cancer Development and Therapy Response. Cancers.

[93] Weersma R.K., Zhernakova A., Fu J. (2020). Interaction between drugs and the gut microbiome. Gut.

[94] Phipps O., Al-Hassi H.O., Quraishi M.N., Kumar A., Brookes M.J. (2020). Influence of Iron on the Gut Microbiota in Colorectal Cancer. Nutrients.

[95] Keeler B.D., Simpson J.A., Ng O., Padmanabhan H., Brookes M.J., Acheson A.G., Banerjea A., Walter C., Maxwell-Armstrong C., Williams J. (2017). Randomized clinical trial of preoperative oral versus intravenous iron in anaemic patients with colorectal cancer. BJS.

[96] Phipps O., Al-Hassi H., Quraishi M., Dickson E., Segal J., Steed H., Kumar A., Acheson A., Beggs A., Brookes M. (2021). Oral and Intravenous Iron Therapy Differentially Alter the On- and Off-Tumor Microbiota in Anemic Colorectal Cancer Patients. Cancers.

[97] Camaschella C. (2015). Iron deficiency: New insights into diagnosis and treatment. Hematology.

[98] Actor J.K., Hwang S.-A., Kruzel M.L. (2009). Lactoferrin as a Natural Immune Modulator. Curr. Pharm. Des..

[99] Bokkhim H., Bansal N., Grøndahl L., Bhandari B. (2016). In-vitro digestion of different forms of bovine lactoferrin encapsulated in alginate micro-gel particles. Food Hydrocoll..

[100] Dix C., Wright O. (2018). Bioavailability of a Novel Form of Microencapsulated Bovine Lactoferrin and Its Effect on Inflammatory Markers and the Gut Microbiome: A Pilot Study. Nutrients.

[101] Knights D., Lassen K.G., Xavier R.J. (2013). Advances in inflammatory bowel disease pathogenesis: Linking host genetics and the microbiome. Gut.

[102] Lee T.W., Kolber M.R., Fedorak R., Van Zanten S.V. (2012). Iron replacement therapy in inflammatory bowel disease patients with iron deficiency anemia: A systematic review and meta-analysis. J. Crohn’s Colitis.

[103] Rizvi S., E Schoen R. (2011). Supplementation With Oral vs. Intravenous Iron for Anemia With IBD or Gastrointestinal Bleeding: Is Oral Iron Getting a Bad Rap?. Am. J. Gastroenterol..

[104] Lee T., Clavel T., Smirnov K., Schmidt A., Lagkouvardos I., Walker A., Lucio M., Michalke B., Schmitt-Kopplin P., Fedorak R. (2016). Oral versus intravenous iron replacement therapy distinctly alters the gut microbiota and metabolome in patients with IBD. Gut.

